# Synergistic antibacterial activity and prevention of drug resistance of daptomycin combined with fosfomycin against methicillin-resistant *Staphylococcus aureus*

**DOI:** 10.1128/aac.01609-24

**Published:** 2025-06-18

**Authors:** Qin Ai, Sailan Wang, Ziyan Chen, Shuai Zheng, Zaixing Chen, Na Zhang, Yaowen Li, Huiping Liu, Yanyan Liu, Jiabin Li, Xiaohui Huang

**Affiliations:** 1 Department of Basic and Clinical Pharmacology, School of Pharmaceutical Sciences, Anhui Medical University 12485, Hefei, China; 2 Anhui Province Key Laboratory of Major Autoimmune Diseases, School of Pharmaceutical Sciences, Anhui Institute of Innovative Drugs, Anhui Medical University 12485, Hefei, China; 3 Department of Infectious Diseases & Anhui Center for Surveillance of Bacterial Resistance, The First Affiliated Hospital of Anhui Medical University 36639, Hefei, China; 4 Anhui Province Key Laboratory of Infectious Diseases & Institute of Bacterial Resistance, Anhui Medical University 12485, Hefei, China; The Peter Doherty Institute for Infection and Immunity, Melbourne, Victoria, Australia

**Keywords:** methicillin-resistant *Staphylococcus aureus*, daptomycin, fosfomycin, antibiotics resistance

## Abstract

The combination of daptomycin and fosfomycin is attracting attention due to the rising resistance observed in methicillin-resistant *Staphylococcus aureus* (MRSA). Most studies indicate that this combination exhibits synergistic or additive antimicrobial activity against MRSA. However, its capacity to prevent resistance remains uncertain. In this study, we first investigated the antibacterial effect of daptomycin and fosfomycin on MRSA, followed by predicting MRSA resistance under the influence of different drugs using a resistance mutation selection window. Subsequently, we conducted preliminary analyses of drug resistance gene mutations in resistance mutants through gene sequencing technology. Additionally, we established an *in vitro* biofilm infection model to explore the internal tolerance associated with phenotypic alterations in biofilms and assess the combination’s ability to prevent drug resistance. Combination drug sensitivity experiments demonstrated that daptomycin and fosfomycin synergistically enhanced antimicrobial effects against the tested strains. This combination reduced the mutant prevention concentration of each drug and narrowed the selection window for drug-resistant mutations. Sequencing results indicated that specific resistance genes were mutated in the single-drug mutants, while no mutations were detected in the combination. Furthermore, the combination exhibited stronger inhibition and removal of biofilm compared to single agents. In conclusion, the daptomycin-fosfomycin combination displays a synergistic antibacterial effect against MRSA and shows enhanced capacity to prevent bacterial resistance, likely attributed to its ability to inhibit resistance gene mutations and exhibit superior anti-biofilm activity.

## INTRODUCTION

Methicillin-resistant *Staphylococcus aureus* (MRSA) is a prevalent multidrug-resistant bacterium encountered in clinical settings. Its resistance to almost all antibiotics currently available, including vancomycin, presents a significant threat to clinical care (1, 2), emphasizing the urgent need for new treatment alternatives. Increasingly, studies suggest that antimicrobial drug combinations may serve as effective therapeutic strategies, offering broader antimicrobial activity and the potential to delay or prevent the emergence of drug-resistant subpopulations of pathogenic microorganisms (3).

Daptomycin is the inaugural lipopeptide antibiotic to receive approval, demonstrating distinct advantages in addressing MRSA-related skin and soft tissue infections, along with bacteremia and infective endocarditis (4). However, during the clinical application of daptomycin, pathogen resistance to this antibiotic has continuously increased (5), with cases of drug resistance reported internationally (6). Consequently, there is a need for additional antimicrobial agents or combinations that may provide valuable insights when identifying severe *S. aureus* infections (7). Fosfomycin has demonstrated a synergistic effect when used alongside various antimicrobial agents (8, 9). Additionally, studies have shown that the combination of daptomycin and fosfomycin exhibits a synergistic effect on MRSA and may be a favorable option for treating severe MRSA infections (10, 11). However, most current research has focused on the antimicrobial activity of these combinations, with limited investigation into their effects on bacterial resistance. As drug resistance progresses, the effectiveness of combination therapies relies not only on decreasing the overall bacterial count but also on suppressing resistant subpopulations.

The resistance mutation selection window (MSW) is an interval within which drug-resistant bacteria are highly susceptible to enrichment and above or below which resistance is less likely to occur (12). The mutant prevention concentration (MPC) and MSW serve as methods for predicting bacterial resistance to antimicrobial drugs *in vitro*, providing a new approach and reference basis for suppressing bacterial drug resistance (13). Furthermore, it has been suggested that low-concentration combination therapy can hinder the selection of drug-resistant mutants by either narrowing or eliminating the MSW (14). The width of the mutant selection window, referred to as the Selection Index (SI), is defined as the ratio of the MPC to the Minimum Inhibitory Concentration (MIC). Thus, we considered measuring MPC and SI to preliminarily predict the ability of the antibiotic combination to limit the evolution of bacterial drug-resistant subpopulations and determine if this impact correlates with *in vitro* antimicrobial synergism.

The occurrence of bacterial resistance is complex. Bacteria can develop drug resistance through the classical pathway of acquiring exogenous resistance genes or chromosomal mutations (15, 16). On the other hand, changes in bacterial phenotype can trigger non-classical resistance mechanisms, leading to internal tolerance (17). Although resistance mutations in daptomycin- or fosfomycin-resistant MRSA have been reported (18, 19), the occurrence of resistance mutations under this specific combination of antibiotics remains unclear. The presence of mutants under single agent and combination during MPC measurements offers the possibility of probing the occurrence of resistance mutations. In addition, biofilms can reduce antibiotic effectiveness, preventing or lowering the concentration of the drug that reaches the bacteria, and creating an environment that may foster the development of resistance. This can lead to persistent infection and resistance spread, an auxiliary pathway for resistance enhancement.

In summary, this study first evaluated the antimicrobial efficacy of the daptomycin-fosfomycin combination against MRSA and assessed its ability to prevent resistance. Based on these findings, we explored the potential mechanisms by which the antibiotic combination limits resistance, encompassing both classical and non-classical forms. Additionally, we validated its antimicrobial efficacy *in vivo*.

## MATERIALS AND METHODS

### Bacterial isolates and mice

In this study, three clinical isolates of methicillin-resistant *Staphylococcus aureus* NO.5, NO.6, and NO.7 from the First Affiliated Hospital of Anhui Medical University were selected as experimental strains. The reference strain ATCC43300 was provided by the Drug Resistance Monitoring Centre of Anhui Province. All strains were identified by the automated VITEK-2 system (BioMerieux, France). Fifty 6-week-old female BALB/C rats weighing 22–24 g were purchased from Pizhou Dongfang Breeding Co., Ltd. (Jiangsu, China).

### Antimicrobials and media

Daptomycin and fosfomycin were purchased from the China Food and Drug Administration and Research Institute (Beijing, China). All isolates were cultured in Muller-Hinton agar (MHA) and Muller-Hinton broth (CAMHB) (Oxoid, England). Additionally, a final concentration of 50 mg/L Ca^2+^ was supplemented for the detection of daptomycin sensitivity, while 25 mg/L glucose-6-phosphate was added for the detection of fosfomycin sensitivity.

### Determination of antimicrobial susceptibility

The minimum inhibitory concentrations (MICs) of daptomycin and fosfomycin against the experimental strains were determined using broth microdilution and agar dilution methods, following the guidelines of the Clinical and Laboratory Standards Institute (CLSI, 2022). The ATCC 43300 strain was employed as a quality control reference. The inoculum volume of the bacterial suspension was adjusted to 5 × 10^5^ CFU/mL and incubated at 37°C for 18–24 hours. The MIC was identified as the lowest concentration where visible colony growth was absent. The experiment was conducted in triplicate.

### Synergy testing by the chequerboard assay

The *in vitro* activity of antibiotic combinations was determined using the micro broth checkerboard method (20). The antibiotic concentrations used in combination were based on a predetermined MIC for each strain in order to test a range consisting of four dilutions below the MIC and two dilutions above the MIC. The bacterial suspension was inoculated at approximately 5 × 10^5^ CFU/mL. Fractional inhibitory concentration index (FICI) was used to evaluate synergism: FICI = (MIC of drug A combination/MIC of drug A alone) + (MIC of drug B combination/MIC of drug B alone). FICI values were interpreted as follows: {less than or equal to} 0.5 = synergism; >0.5 but {less than or equal to} 1 = additive; >1 but {less than or equal to} 4 = no interaction; >4 = antagonism.

### *In vitro* time-killing curves

Preliminary evaluation of the *in vitro* activity of daptomycin and fosfomycin alone and in combination was assessed using time-kill assays. Briefly, the experiment was divided into four groups: blank control group, 1/2 MIC daptomycin, 1/2 MIC fosfomycin, and 1/2 MIC daptomycin + 1/2 MIC fosfomycin combination group. The bacterial suspension and each group of drugs were added to the test tubes to achieve a final concentration of 5 × 10^5^ CFU/mL, and then the tubes were incubated with shaking at 37 ℃ for 24 hours. Aliquots were taken at 0, 2, 4, 8, 12, and 24 hours for colony counting. Additivity and synergism were defined as reductions of 1–2 log_10_ CFU/mL and ≥2 log_10_ CFU/mL, respectively, of the combination drug compared to the most active single drug in the combination (21).

### MPC alone or in combination

The MPCs of daptomycin, fosfomycin, and their combination in the strains were determined using the agar plate dilution method reported in the literature (22). Briefly, the antimicrobials were admixed into MHA plates. A bacterial suspension was concentrated to 10^10^ CFU/mL and inoculated onto drug-containing plates with sequential twofold increasing concentrations. Concentrations of single drugs ranged from 1 × MIC to 64 × MIC. Different concentrations of daptomycin and fosfomycin, ranging from 0.5 × MIC to 8 × MIC, were prepared using twofold serial dilutions based on MIC and FICI values. A range of combination ratios (daptomycin : fosfomycin, from 8:0.5 to 0.5:8) was designed for use in combined MPC studies. The plates were incubated for 72 hours, and the concentration of the first plate that did not show growth was considered the MPC. For each strain, colonies grown at the highest daptomycin or fosfomycin concentration and on the highest combined group were passaged three times on drug-free agar and then inoculated in medium similar to the concentration of MPC to be identified as a drug-resistant mutant strain and saved for further testing.

### Characterization of resistance

Resistance mutants obtained in the resistance mutation selection window study under single and combination were sequenced and compared to the parent strain sequence. Screening for possible mechanisms of daptomycin resistance by polymerase chain reaction (PCR): mutations in the mprF and cls2 genes. The fosfomycin-related resistance genes fosB, murA, glpT, and uhpT were also amplified by PCR . Nucleotide and amino acid sequences were analyzed using the sequence analysis software BLAST (https://blast.ncbi.nlm.nih.gov/Blast.cgi) and SnapGene Viewer 6.0.2 to compare the occurrence of mutations.

### Biofilm growth curves

The microdrop plate method described by Yu et al. (23) was used to quantitatively measure the formation of biofilms, and the method was improved. A 0.2 mL bacterial suspension with a final concentration of 10^7^ CFU/mL was added to 96-well plates, with culture medium serving as a blank control. The plates were incubated at 37°C for 6, 12, 24, 36, 48, and 72 hours. The amount of biofilm was detected by crystal violet staining. After the incubation was completed, the medium was discarded and washed with PBS to remove planktonic bacteria. Then methanol was added to each well, and after fixation, the methanol was discarded and washed with PBS. To each well, 0.1% crystal violet staining was added, washed, and dried with PBS, ethanol solution was added, and then the absorbance was measured at 590 nm. Each assay was performed in triplicate three times. The biofilm growth curve was plotted.

### Inhibition of biofilms by antibiotics

As previously mentioned, crystal violet staining was used to analyze the effects of single or combination drugs on MRSA biofilms. Briefly, MRSA isolates were inoculated into 96-well plates containing CAMHB and 1/2 MIC daptomycin, 1/2 MIC fosfomycin, or 1/2 MIC daptomycin + 1/2 MIC fosfomycin, with positive and negative controls. After 48 hours of incubation, the remaining biofilm biomass was determined by crystal violet staining, and its OD value was measured at 590 nm. The experiment was repeated three times. The evaluation indexes were expressed using the following formula:


%BI={(ODC−ODT )/ODC}×100

%BI is the percentage of inhibition of biofilm formation; ODC is the absorbance at 590 nm for the non-drug-treated group; ODT is the absorbance at 590 nm for the drug-treated group.

### Clearance of mature biofilms by antibiotics

The removal of biofilm by the drug was measured by incubating cultures in 96-well plates for 48 hours to allow biofilm formation to mature, followed by administration of the drug as described above. After 24 hours of drug treatment, the remaining biofilm biomass was assessed using crystal violet staining. The experiment was repeated three times, maintaining the same evaluation criteria as before.

### Scanning electron microscope observation of morphology

A 6-well plate was selected, and a sterile coverslip was placed in each well and incubated with bacterial solution for 48 hours to allow the biofilm to form on the slide. The slide was then transferred to a new 6-well plate, and the drug concentration (as previously defined for biofilm measurements) was added. The plates were incubated at 37°C for 24 hours. The medium was discarded and washed three times with sterile PBS buffer. Add 2 mL of 2.5% glutaraldehyde and incubate overnight at 4°C for fixation. After fixation, remove the 2.5% glutaraldehyde and add 2% osmium tetroxide, allowing it to fix the sample for 2 hours. Perform gradient dehydration using 50%, 75%, 80%, 95%, and 100% ethanol sequentially, with each dehydration step lasting 15 minutes. Dry the samples using a critical point dryer and then coat them with gold powder using an ion sputtering instrument under vacuum conditions. Observed under scanning electron microscope with 10,000 times magnification.

### Mouse bacteremia model

Fifty female BALB/c mice were used for the experiment. All animals were acclimatized and fed for 1 week, after which they were injected intravenously into the tail vein with 0.1 mL of a bacterial suspension at a concentration of 1 × 10^9^ CFU/mL (24). Six mice were randomly selected for humane euthanasia 24 hours after the injection as a baseline control. The remaining mice were randomly divided into four groups: solvent control (*n* = 14), daptomycin (*n* = 10), fosfomycin (*n* = 10), and daptomycin-fosfomycin (*n* = 10). The treatment regimens for each group were as follows: 50 mg/kg daptomycin administered every 24 hours, 100 mg/kg fosfomycin administered every 4 hours, or a combination of 50 mg/kg daptomycin and 100 mg/kg fosfomycin. Solvent control groups received equivalent volumes of PBS. In the mouse model, the dosing regimen of 50 mg/kg/day for daptomycin approximates the human AUC_0-24_ target exposure achieved with 6 mg/kg/day (25). For fosfomycin, a dosing regimen of 100 mg/kg every 4 hours was selected, as it produces a mean peak serum concentration of 132 ± 20 mg/L and a mean AUC_0-24_ of 504 mg·h/L, closely mirroring the levels achieved in humans with a 4 g infusion every 8 hours (26). Treatment lasted 1 day. The survival of mice was observed every 12 hours and recorded in time. On day 4, the mice were anesthetized, blood was collected from the retro-orbital vein, and the spleen was aseptically removed. Both the blood and spleen were assessed for colonization.

### Statistical analysis

All statistical analyses were performed with GraphPadPrism, version 9.0 (GraphPad Software, Inc., San Diego, CA, United States). Data were evaluated with a one-way ANOVA. *P*-values < 0.05 were deemed statistically significant.

## RESULTS

### Minimal inhibitory concentration and checkerboard assay

The MIC and FICI values of all tested strains against the antimicrobial drugs are shown in Table 1. The results indicate that the MIC of daptomycin for four strains was 0.5 mg/L, while the MIC of fosfomycin varied between 2 and 16 mg/L. No bacterial strains demonstrated resistance to either daptomycin or fosfomycin. The checkerboard assay revealed that the FICI values for the combination of daptomycin and fosfomycin against all experimental strains were less than or equal to 0.5, suggesting a synergistic effect on MRSA strains.

**TABLE 1:** MICs and FICI of antimicrobial against four strains^*a*^

Isolates	MIC(mg/L)		FICI
	DAP	FOF	
ATCC 43300	0.5	8	0.5
NO.5	0.5	8	0.5
NO.6	0.5	2	0.375
NO.7	0.5	16	0.5

^*a*^ MIC, minimum inhibitory concentration; DAP, daptomycin; FOF, fosfomycin; FICI, fractional inhibitory concentration index.

### *In vitro* time-killing curves

**Fig 1:**
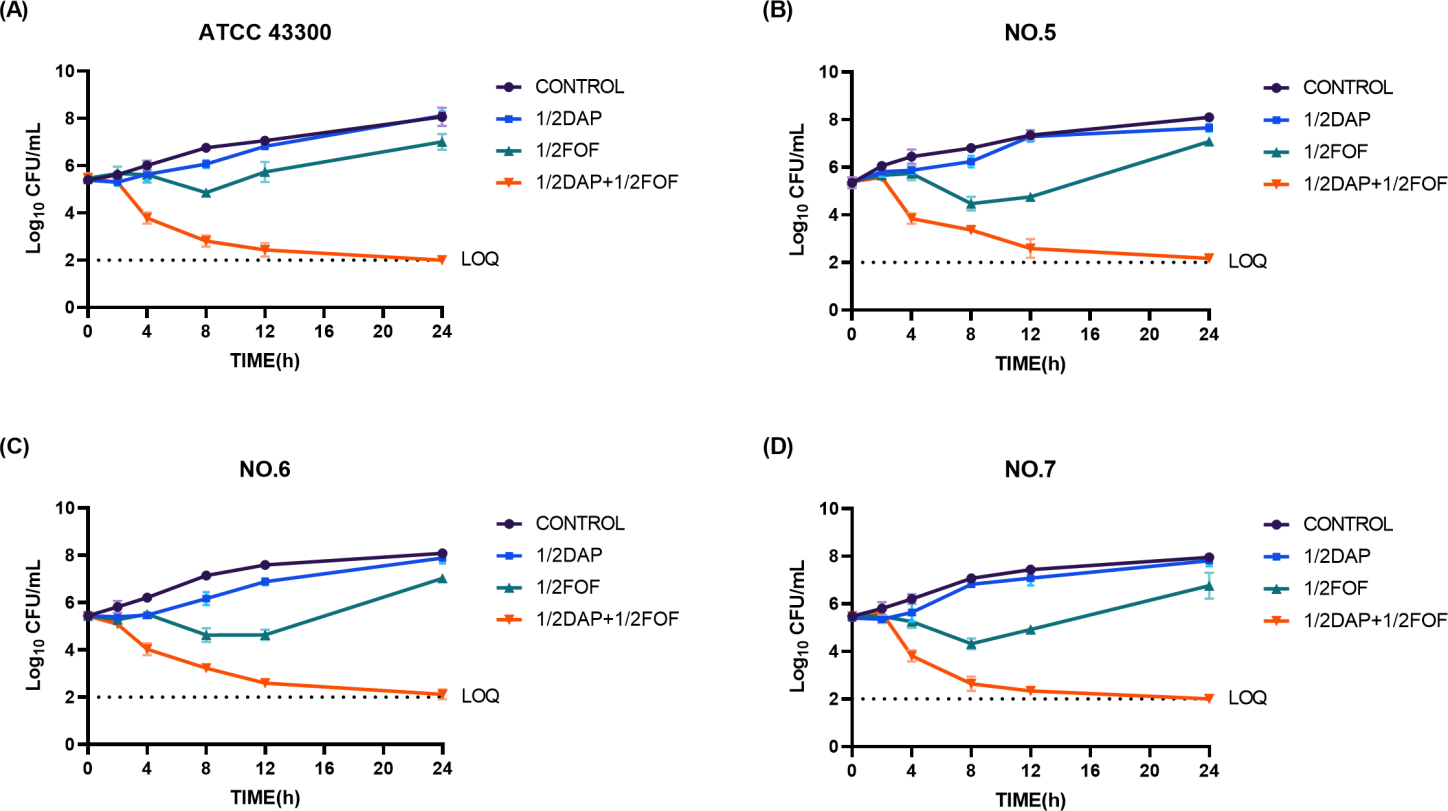
Static-concentration time-kill studies displaying the activity of daptomycin, fosfomycin, and their combination against MRSA. ATCC 43300 (**A**), NO.5 (**B**), NO.6 (**C**), and NO.7 (**D**). CFU, colony-forming units; DAP, daptomycin; FOF, fosfomycin; Control: no drug; 1/2, 1/2 × MIC; LOQ: quantification limit.

Fig. 1 displays the results of the time sterilization curves. For all strains, the 1/2MIC daptomycin treatment group had no bactericidal effect, and its colony count was very close to that of the control group throughout the action. In the 1/2 MIC fosfomycin treatment group, bacterial killing ranged from 1 to 2 log_10_ at 4–8 hours. However, bacterial regrowth was observed after 8–12 hours, with colony counts exceeding the initial inoculum levels by 24 hours. In contrast, the combination of the two drugs demonstrated superior and long-lasting bactericidal activity. Additionally, the number of colonies decreased by more than 2 log_10_ CFU/mL at 24 hours compared to the best-performing single agent, suggesting a synergistic antimicrobial effect of daptomycin in combination with fosfomycin.

### Mutant prevention concentration and mutant selection window

As shown in Table 2, the MPC of all three isolates with daptomycin alone was 8.0 mg/L, and the SI was 16. In combination with fosfomycin, the MPC decreased to 1 mg/L, and the SI dropped to 2. The MPC value of fosfomycin alone ranged from 64 to 256 mg/L, with an SI of 16–32. After combining with daptomycin, the MPC was reduced to 4–64 mg/L, and the SI decreased to 2–4. Compared with daptomycin or fosfomycin alone, the combination of both drugs reduced the MPC and narrowed the window of selection for resistance mutations.

**TABLE 2:** Effects of DAP and FOF alone and in combination on MPC and SI in MRSA^*a*^

Isolates	DAP		FOF		DAP + FOF			
	MPC	SI	MPC	SI	MPC_DAP_	SI_DAP_	MPC_FOF_	SI_FOF_
NO.5	8 mg/L	16	128 mg/L	16	1 mg/L	2	16 mg/L	2
NO.6	8 mg/L	16	64 mg/L	32	1 mg/L	2	4 mg/L	2
NO.7	8 mg/L	16	256 mg/L	16	1 mg/L	2	64 mg/L	4

^*a*^ DAP, daptomycin; FOF, fosfomycin; MPC, mutant prevention concentration; SI, mutant selection indexes.

### Resistance genes

Sequencing comparison between the resistant mutants and the parental strains yielded the results shown in Tables 3 and 4. Among the daptomycin monoresistant mutant strains, mutations in the mprF gene were identified in strains NO.5-DM and NO.6-DM. Sequencing revealed nucleotide substitutions at position 905(C→T) in strain NO.5-DM and position 1033(A→G) in strain NO.6-DM, both of which resulted in amino acid changes in mprF. Additionally, a mutation in the cls2 gene was observed in strain NO.5-DM. For the fosfomycin monoresistant mutant strains, mutations in the uhpT gene were detected in strains NO.5-FM and NO.7-FM. Sequencing showed nucleotide substitutions at position 932(C→T) in strain NO.5-FM and position 499(A→G) in strain NO.7-FM, leading to amino acid changes in mprF. Furthermore, a mutation in the murA gene was identified in strain NO.6-FM, caused by a nucleotide deletion at position 581. Notably, no mutations in these genes were detected in the resistant mutant strains treated with a combination of daptomycin and fosfomycin. No target fragment of fosB was amplified.

**TABLE 3:** Sequencing results of daptomycin-associated resistance genes^*a*^^,^^*b*^

Isolates	Resistance gene	
	mprF	cls2
NO.5-DM	905 C > T (Ala302Val)	1298 A > G (Asn433Ser)
NO.5-DFM	-	-
NO.6-DM	1033 A > G (Thr345Ala)	-
NO.6-DFM	-	-
NO.7-DM	-	-
NO.7-DFM	-	-

^*a*^ +, Presence of mutations, - No mutations.

^*b*^ NO.5-DM, NO.6-DM, and NO.7-DM are the corresponding daptomycin-resistant mutants obtained from MPC studies with daptomycin alone; NO.5-DFM, NO.6-DFM, and NO.7-DFM are the corresponding combination group resistance mutants obtained from MPC studies of daptomycin in combination with fosfomycin.

**TABLE 4:** Sequencing results of fosfomycin-associated resistance genes^*a*^^,^^*b*^

Isolates	Resistance gene		
	murA	uhpT	glpT
NO.5-FM	-	932 C > T (Ala311Val)	-
NO.5-DFM	-	-	-
NO.6-FM	581del A	-	-
NO.6-DFM	-	-	-
NO.7-FM	-	499 A > G (Arg167Gly)	-
NO.7-DFM	-	-	-

^*a*^ +, Presence of mutations, - No mutations.

^*b*^ NO.5-FM, NO.6-FM, and NO.7-FM are the corresponding fosfomycin-resistant mutants obtained from MPC studies with fosfomycin alone; NO.5-DFM, NO.6-DFM, and NO.7-DFM are the corresponding combination group resistance mutants obtained from MPC studies of daptomycin in combination with fosfomycin.

### Biofilm growth curves

**Fig 2:**
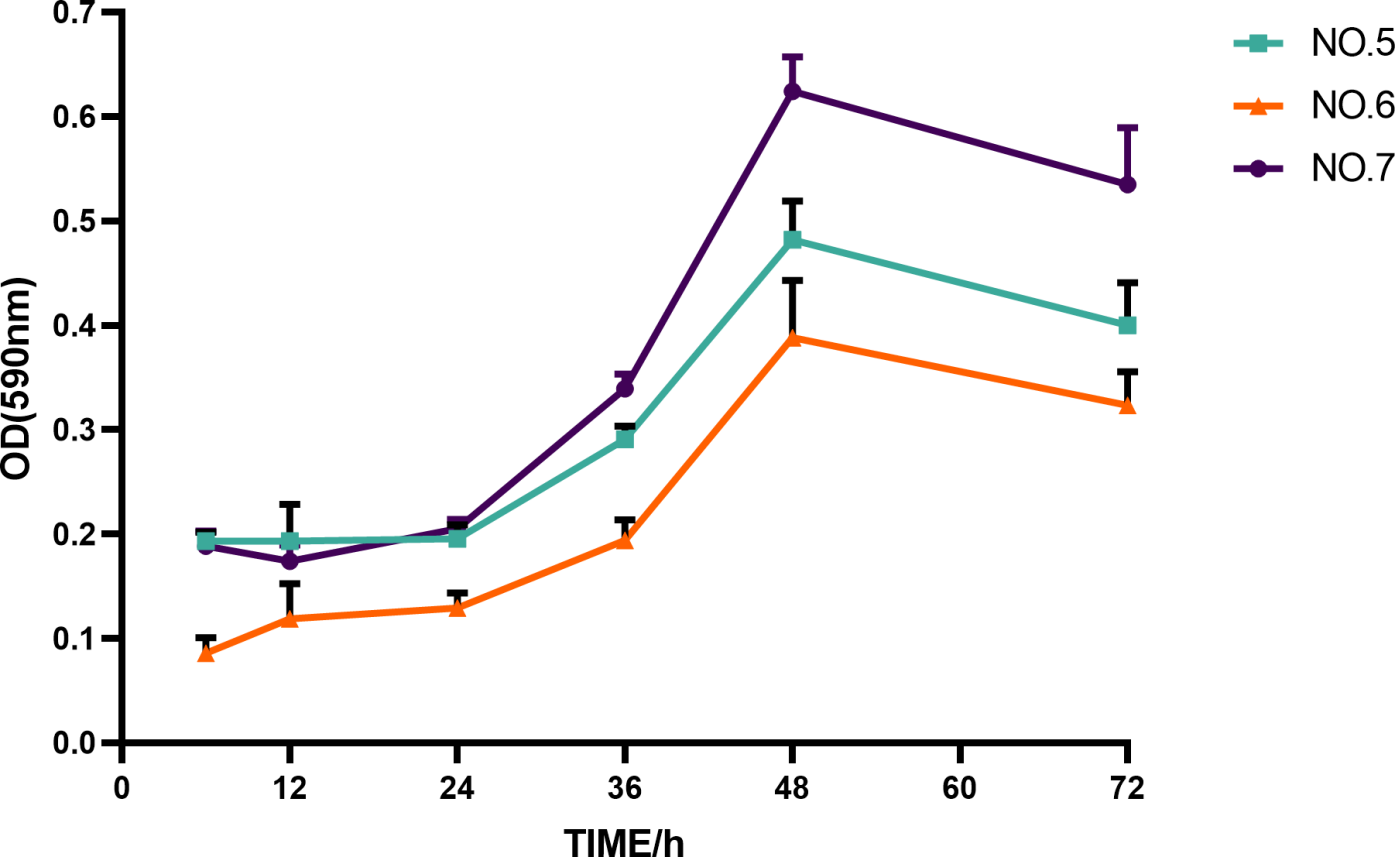
Growth curves of MRSA biofilm substrates. Abbreviations: OD, optical density.

All three isolates had strong biofilm formation ability, and the curves of the formed biofilm matrix are shown in Fig. 2. It can be observed from the figure that the biofilm formation of the strain increased gradually during the first 48 hours and reached a maximum value at the 48 hour time point, after which it started to disperse. Therefore, the *in vitro* biofilm formation time in this experiment was 48 hours.

### Inhibition and clearance of biofilms by antibiotics

**Fig 3:**
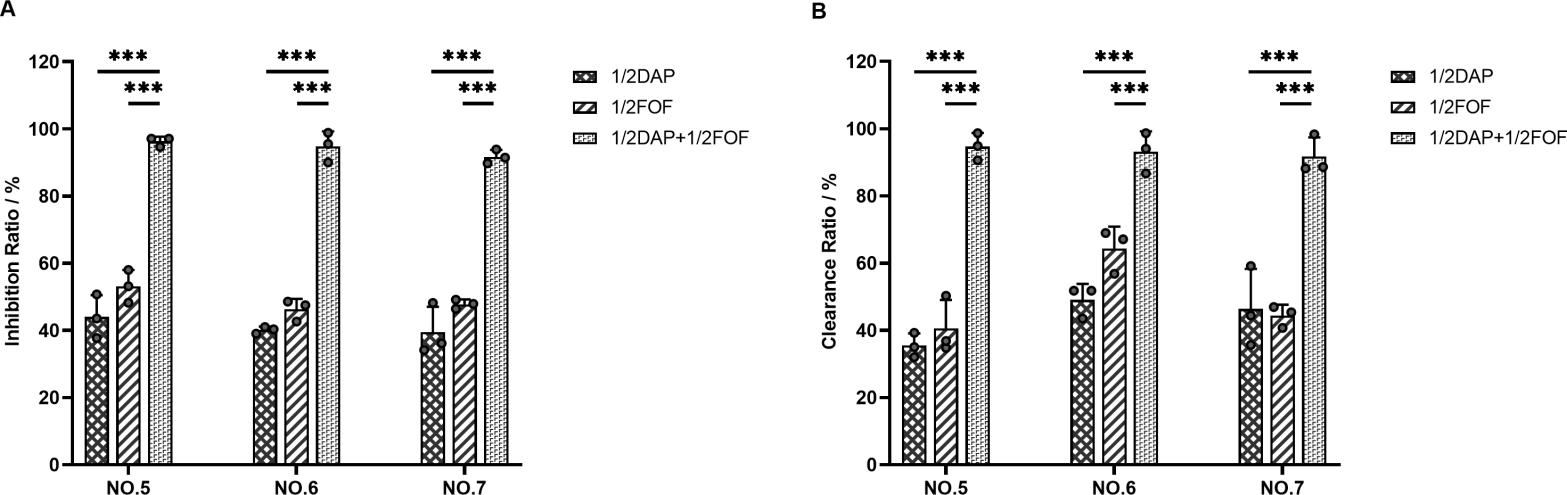
Inhibitory effect of daptomycin and fosfomycin on biofilm (A) and clearance of mature biofilm (B). Data represent the average of three independent experiments (mean ± SD). *** , *P*-value ≤0.001. DAP, daptomycin; FOF, fosfomycin; Control: no drug; 1/2, 1/2 × MIC.

As shown in Fig. 3A, both daptomycin and fosfomycin inhibited MRSA biofilm growth across the three experimental strains to varying degrees, whether used alone or in combination. However, when the two drugs were combined, the inhibition rate was significantly higher and more effective in preventing biofilm formation. Additionally, as illustrated in Fig. 3B, the combination demonstrated a significantly greater efficacy in removing mature biofilms compared to either daptomycin or fosfomycin alone.

### SEM analysis

**Fig 4:**
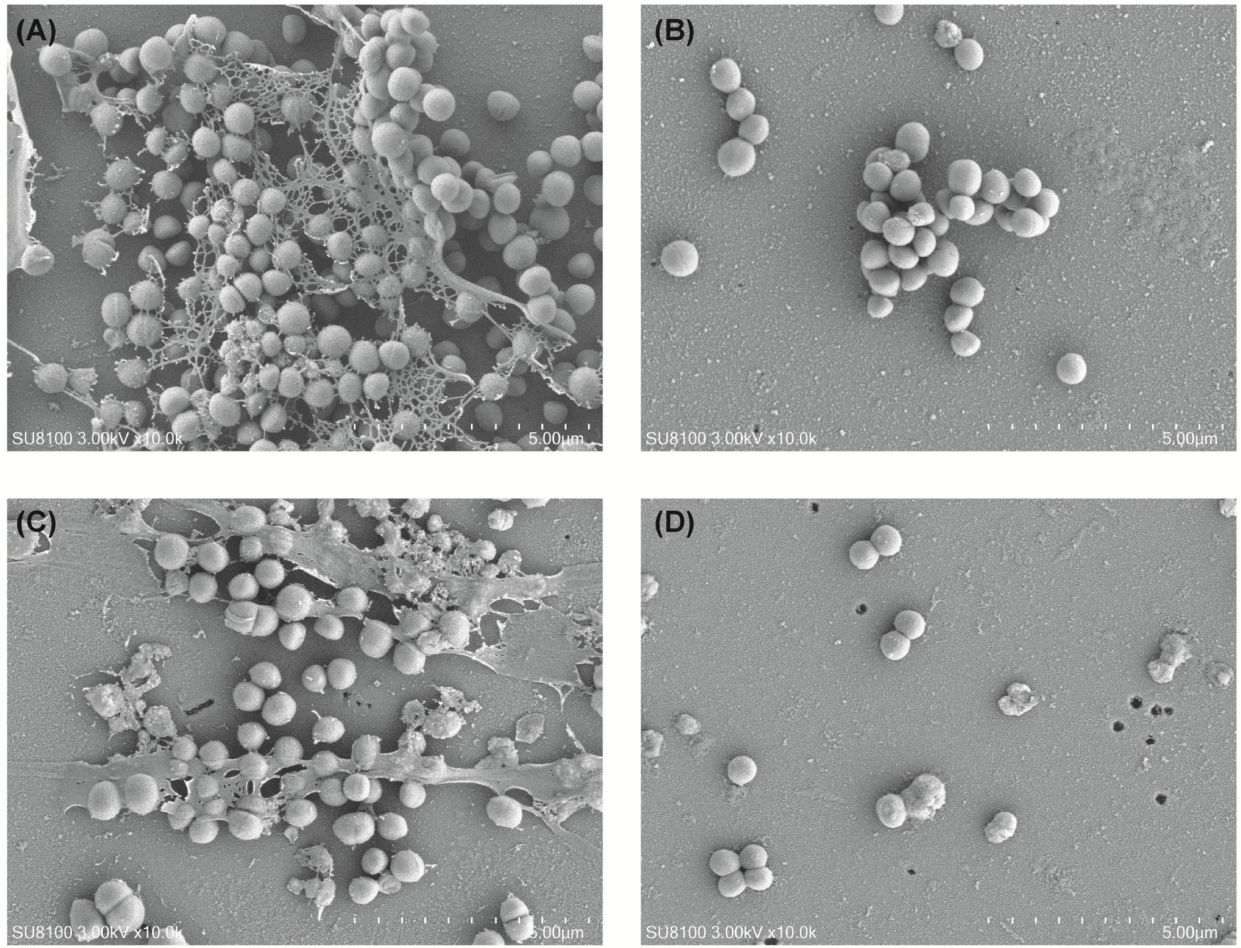
Scanning electron microscopy of biofilm morphology in each group. Control (**A**), Daptomycin (**B**), Fosfomycin (**C**), Daptomycin-fosfomycin combination (D). DAP, daptomycin; FOF, fosfomycin; Control, no drug.

The results are shown in Fig. 4. In the control group, a substantial amount of extracellular secretion adhered to the carrier surface, with numerous MRSA bacteria present both inside and outside the biofilm. In the daptomycin group, extracellular secretion was significantly reduced compared to the control group although bacteria were still visible in clumps. In the fosfomycin group, MRSA adherence was significantly diminished relative to the control, but residual extracellular secretion remained on the carrier surfaces, forming a biofilm structure. In contrast, the daptomycin-fosfomycin combination resulted in a marked reduction in both bacterial adherence and extracellular secretion, with no visible clusters or flaky biofilm present. These findings are consistent with the crystal violet staining results.

### Therapeutic effect on experimental MRSA bacteraemia

**Fig 5:**
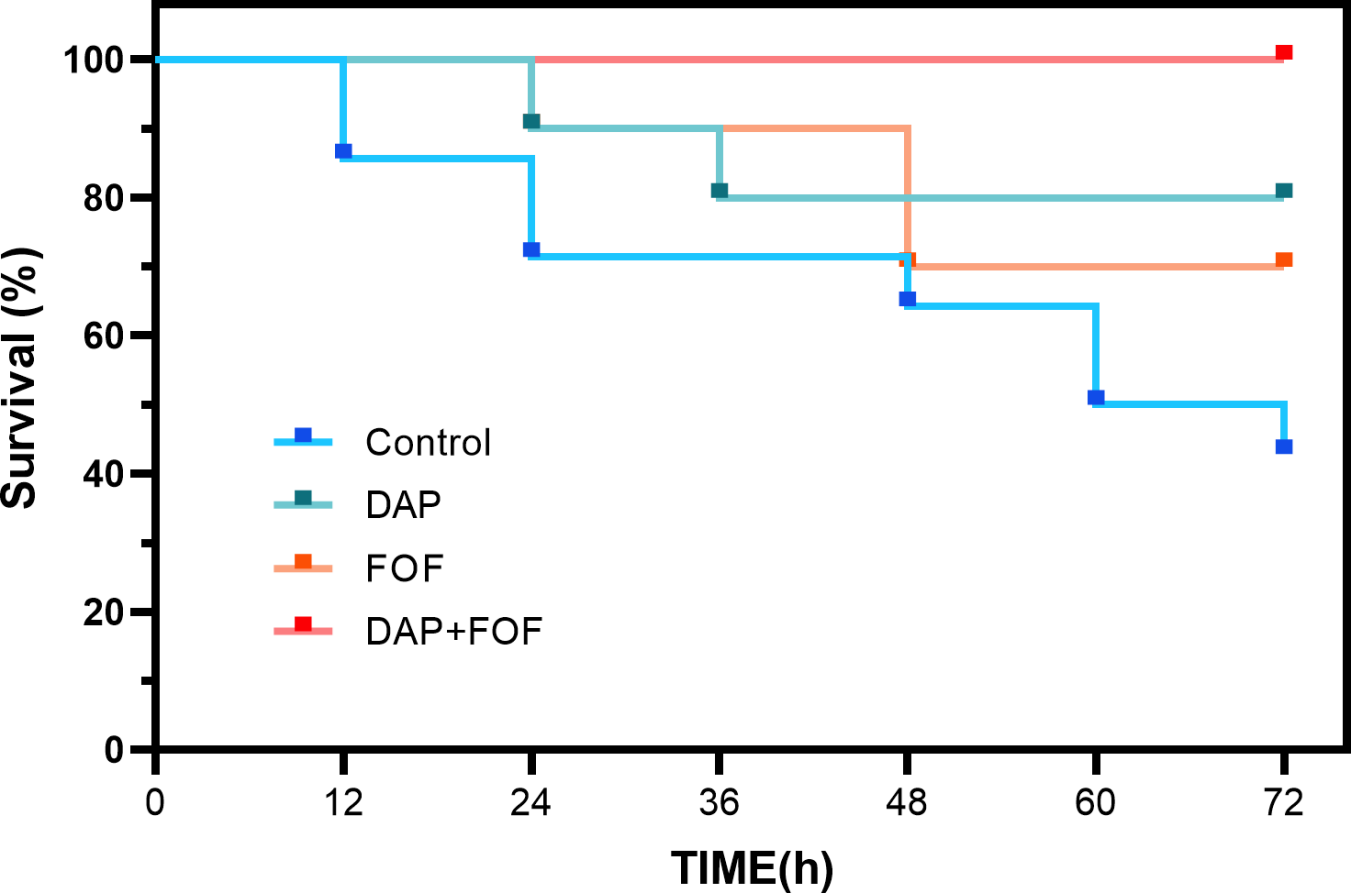
Survival of mice at 72 hours of different treatment regimens in a mouse model of bacteremia infection. DAP, daptomycin; FOF, fosfomycin; Control, no drug.

**Fig 6:**
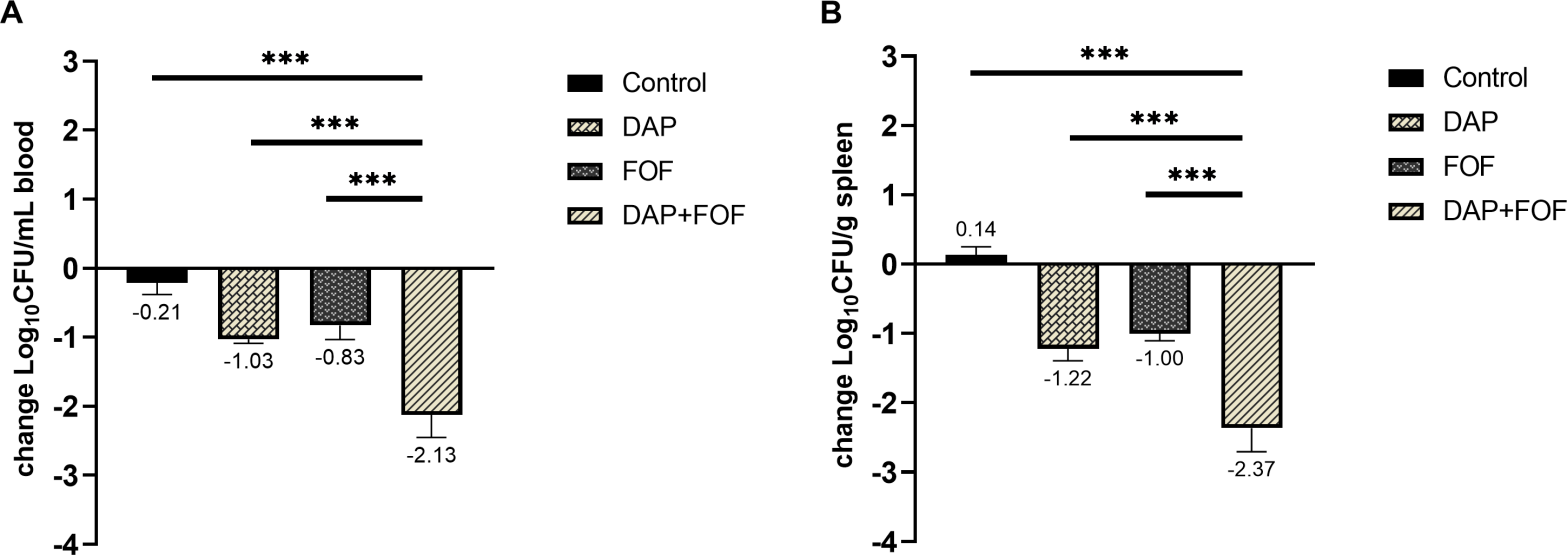
Effect of antimicrobial therapy on the clearance of MRSA strains in blood and spleen. Changes (mean ± s.d.) in blood (**A**) log_10_ CFU/mL and kidney (**B**) log_10_ CFU/g relative to baseline in mice infected with MRSA strains (untreated mice, *n* = 6); control (*n* = 6) and daptomycin, fosfomycin, and combination-treatment groups (*n* = 6 in each group). ***, *P*-value ≤ 0.001. CFU, colony-forming units; DAP, daptomycin; FOF, fosfomycin; Control, no drug.

As shown in Fig. 5, 72 hours post-administration, the survival rate for the solvent control group was 42.86%, while the daptomycin monotherapy group had a survival rate of 80%, the fosfomycin monotherapy group showed 70%, and the combination group achieved a survival rate of 100%. At 24 hours, the blood bacterial load of the mice was 4.69 log_10_ CFU/mL, and the spleen bacterial load was 6.57 log_10_ CFU/g. After 72 hours of treatment, bacterial concentrations in the blood were reduced by 1.03 log_10_ CFU/mL in the daptomycin group, 0.83 log_10_ CFU/mL in the fosfomycin group, and 2.13 log_10_ CFU/mL in the combination group (Fig. 6A). Spleen colony counts decreased by 1.22 log_10_ CFU/g, 1.00 log_10_ CFU/g, and 2.37 log_10_ CFU/g, respectively (Fig. 6B). Compared with monotherapy, the combination increased the survival rate of infected mice and significantly reduced the bacterial load.

## DISCUSSIONS

The FICI values for all the strains in this study and the results of the *in vitro* time-kill curves showed that daptomycin and fosfomycin had synergistic effects on all experimental strains of MRSA. Consistent with this finding, the synergistic antimicrobial effect of the daptomycin-fosfomycin combination against *Staphylococcus aureus in vitro* has been confirmed multiple times (9, 27). However, there is a dearth of studies focusing on the prevention of drug resistance through combination therapies.

The MPC and MSW are crucial for preventing bacterial drug resistance. Evidence from *in vitro* and animal models suggests that MSW values can effectively estimate which drug concentrations can promote resistance development and should be avoided; a narrower MSW indicating greater efficacy in limiting the emergence of bacterial resistance mutation (28–30). For the strains studied, the MPCs of fosfomycin alone were 16–32 times higher than their MICs, indicating a broad MSW for fosfomycin. Similar MPC studies of fosfomycin against *E. coli* (31) and *Pseudomonas aeruginosa* (14) have yielded comparable results. Pharmacokinetic studies of fosfomycin indicate that a single oral dose of 3 g achieves a peak concentration (Cmax) of 26.8 ± 6.4 mg/L (32), whereas a 4 g intravenous infusion achieves a Cmax ranging from 200 to 250 mg/L (33). In this study, some MRSA strains exhibited a Cmax/MPC ratio of less than 1 for fosfomycin, indicating that when fosfomycin is used alone for clinical treatment, the drug concentration at the infection site may fall within the MSW range, thereby promoting the proliferation of fosfomycin-resistant bacteria. The plasma protein binding rate of daptomycin is as high as 90% (34), and the free drug concentration at the routine clinical dose of 6 mg/kg is likely to fall within the selection window for drug-resistant mutations. Moreover, daptomycin exhibits poor tissue penetration, resulting in significantly lower concentrations in bone and other tissues compared to plasma, which greatly increases the risk of drug resistance. Thus, daptomycin alone is insufficient to limit the enrichment of resistant mutants. In this study, the combination of daptomycin and fosfomycin resulted in lower MPC values, even reaching as low as 1/32 of the original, along with a substantial narrowing of the MSW. This reduction is clinically significant for preventing the enrichment of drug-resistant mutants although it does not completely eliminate the selection window for these mutants. Meanwhile, we found that the minimum preventive concentration (MPC) of daptomycin was 1 mg/L when combined with fosfomycin. This concentration falls within the range of daptomycin sensitivity and aligns with the findings of Mishra et al. (35), who demonstrated that the combination of daptomycin and fosfomycin could inhibit the emergence of daptomycin-resistant strains from daptomycin-sensitive parental strains *in vitro*. Additionally, Golikova et al. (36) discovered that combining daptomycin with other antibiotics curtailed the emergence of daptomycin-resistant *S. aureus* mutants. According to MSW theory, increasing a drug’s concentration above the MPC requires a significant dose increase, which may lead to serious toxic side effects in certain organs (22). Furthermore, developing new drugs is a time-consuming process. Therefore, an effective approach is to reduce or eliminate the MSW by combining two antimicrobial agents.

In *Staphylococcus aureus*, several mutations in the mprF and cls2 genes are prevalent in daptomycin-resistant strains isolated from clinical and laboratory settings (37, 38). Point mutations or abnormal expression of these genes may confer resistance to daptomycin. Resistance to fosfomycin can arise from mechanisms such as mutations and overexpression of murA (39), reduced uptake of fosfomycin (40), and expression of modifying enzymes (41). In summary, genetic mutations in resistance-associated genes can diminish the bactericidal efficacy of antibiotics, posing a significant threat to clinical treatment. Therefore, understanding the alterations in drug-resistant mutants is crucial for informing the prudent use of antibiotics and mitigating resistance. In this study, we screened for phenotypically altered drug-resistant bacteria by MSW and amplified and sequenced the parental strain and drug-resistant mutants. Unlike previous studies, we examined not only single-drug resistant mutants but also those under combined effects, comparing them with parental strains and discussing the genetic variation of resistance genes. We identified mutations in the mprF gene in two of the three daptomycin-resistant mutant strains. The results are consistent with recent studies that reported the presence of point mutations in mprF in daptomycin-resistant mutants screened on the basis of MPC (42). We also identified mutations in a single resistance gene in three mutants resistant to fosfomycin. Our sequencing analysis identified distinct mutations in the uhpT gene of two fosfomycin-resistant mutants. Fosfomycin transport in bacteria primarily relies on the glucose-6-phosphate transporter (UhpT) and glycerol-3-phosphate transporter (GlpT). Mutations or base deletions in the genes encoding these transport systems can result in loss of transporter protein function, preventing fosfomycin uptake and conferring resistance. Recent studies have also shown that amino acid substitutions in uhpT can reduce fosfomycin transport efficiency, thereby promoting resistance (40). Additionally, we detected a mutation in murA in one of the mutants, which has been reported to reduce the enzyme’s affinity for fosfomycin, further contributing to resistance (19). Interestingly, none of the mutants obtained under the combined action of daptomycin and fosfomycin in this study had detectable mutations. This finding aligns with Drlica et al. (43), which indicates that when a drug is used alone, bacteria can develop resistance through a single mutation; conversely, with combination therapy, bacteria must acquire resistance to both drugs simultaneously, theoretically occurring at a much lower rate. Therefore, we deduced that co-administration of daptomycin and fosfomycin may inhibit the development of resistance mutations, thereby enhancing the ability to prevent the evolution of resistance. Given the complexity of bacterial resistance mechanisms, more work is needed in the future to identify these mutations as well as other mutations or overexpression of genes that result in reduced microbial activity.

In addition to classical mutations in drug resistance genes, the formation of bacterial biofilms is one of the most common causes of bacterial resistance development. Therefore, this paper also innovatively explores the reasons for the combination of daptomycin and fosfomycin in preventing the development of resistance from the perspective of biofilm, a phenotypic alteration that leads to nonclassical resistance, in a new way. Bacterial biofilms are a key target for bacterial resistance to antibiotics, increasing resistance to environmental stresses, and are up to 1,000 times more resistant to antibiotics compared to the planktonic state (44). Additionally, horizontal gene transfer occurs frequently within biofilms, further contributing to bacterial resistance to antibiotics (45). Due to the inherent characteristics of biofilms, infections linked to biofilms are especially challenging to eliminate and act as reservoirs for the transmission of bacteria and antibiotic resistance genes (46). Daptomycin is known to inhibit bacterial biofilm formation (47). This raises the question: does the combination of two drugs enhance the effect on MRSA biofilms compared to one drug alone, thereby limiting the emergence of resistance? Biofilm biomass measurements clearly indicated that while both daptomycin and fosfomycin exhibited varying degrees of inhibition and clearance, the combination of these drugs significantly enhanced these effects, achieving clearance and inhibition rates of 90% or higher. Simultaneously, the ability of MRSA to form biofilms on both biotic and abiotic surfaces is the primary mechanism by which they develop resistance to antibiotics (48). The extremely high level of bacterial resistance within biofilms, along with the difficulty in removing biofilm structures, contributes to the persistence of drug-resistant bacterial infections, presenting a major clinical challenge in prevention and treatment. However, the daptomycin-fosfomycin combination in this study demonstrated excellent inhibitory and clearance capabilities against biofilms, suggesting it may be a beneficial strategy for preventing the development of drug resistance.

*In vitro* studies have demonstrated that the combination of daptomycin and fosfomycin has a synergistic effect on MRSA; however, it remains uncertain whether it is effective in more complex organisms. Drug activity is often inconsistent between *in vitro* and *in vivo* environments, and a single *in vitro* activity test is insufficient for fully assessing the antimicrobial effects of a drug. Therefore, we conducted a more comprehensive evaluation of the *in vivo* antibacterial effectiveness of daptomycin in conjunction with fosfomycin by creating a mouse model of bacteremia. The results demonstrated that in this model, the combination therapy reduced bacterial load more effectively than monotherapy. Additionally, the combination of daptomycin and fosfomycin has shown effective bacterial killing in various experimental settings, including models of infective endocarditis and osteomyelitis (10, 49). Both *in vitro* and *ex vivo* results suggest that combining daptomycin and fosfomycin is a promising strategy for treating MRSA infections.

We acknowledge several limitations of this study. First, we investigated only three MRSA strains and did not analyze their genetic backgrounds, which may limit the representativeness of our findings. To enhance the generalizability of these findings, future research should incorporate a more comprehensive collection of isolates representing broader genetic diversity, with specific inclusion of fosB-positive strains not evaluated in the current study. Second, we focused on a limited set of common resistance genes and may have overlooked other critical genetic variants. Our findings on pathogenic mutations, based on comparisons between susceptible parental sequences and resistant mutant sequences, require further validation through knockout or targeted mutation experiments. Additionally, we quantified *in vitro* biofilm models using crystal violet staining, but the reproducibility of this method is relatively low. Finally, this study examined the ability of the drug combination to inhibit resistance gene mutations and biofilm formation, focusing on both classical and non-classical mechanisms of resistance. Recent studies suggest that fosfomycin may alter the bacterial metabolome by disrupting key metabolic pathways, such as glycolysis (e.g., inhibition of enolase) and the tricarboxylic acid (TCA) cycle. These metabolic changes increase cardiolipin content and modify membrane charge, thereby enhancing daptomycin’s binding affinity to bacterial cell membranes. Additionally, these disturbances increase membrane fluidity, facilitating the embedding of daptomycin and enhancing its antibacterial activity. From a metabolomic perspective, these findings suggest a potential mechanism by which the combination of fosfomycin and daptomycin may prevent the emergence of resistance (35, 50). Therefore, further investigation into the role of metabolic adaptations in resistance development is warranted.

### Conclusions

In this study, we demonstrated the synergistic antibacterial effect of combining daptomycin and fosfomycin against MRSA and provided some important insights into the potential mechanistic basis for this combination in preventing the evolution of MRSA resistance. However, due to the limited number of strains examined, further validation using a more extensive and genetically diverse collection of clinical isolates is required to confirm the generalizability of these findings.

## Data Availability

The sequence of strains has been deposited in GenBank under accession numbers PQ298983-PQ299000 (mprF and cls2 gene related sequences), PQ285482-PQ285499 (murA and uhpT gene related sequences), and PQ299001-PQ299009 (glpT gene related sequences).
